# 

**Published:** 1917-03-31

**Authors:** 


### CONTENTS.

PAGE

**A Ministry of Health** ... ... ... ... ... 507

**What is Truth?** ... ... ... ... ... ... 508

**Hospital and Institutional News** ... ... ... ... 509

A New Departure at the Middlesex Hospital—Military Hospital Expansion in Wales—Constitutional Changes at the Royal Sussex County Hospital—Resident Medical Officers at Private War Hospitals—Progress of the Leicester Saturday Hospital Society—St. Bartholomew's Hospital and the Venereal Crusade—Hospital Medical Officers and Dying Declarations—The Doctor's Responsibility in these Cases—The Liverpool Hahnemann Hospital—The Deeds of Selection Committees—The Ethics of Hospital Dinners in Wartime—A Devonshire Hospitals Fund—The Needs of the Civil Population—"Have You Any Potatoes?"—A Staff and Soldiers at the Spade—The Hospital Standard and the Domestic Staff—Rice Flour and Maize Flour for Bread—The Killing of Asylum Cows—Hospitals Shareholders in Drapery Establishment—The Hour of the Annual Meeting—Munition Girls and the Wounded—Nurses' Uniforms for Health Visitors—"Heroin" and the Medical Profession—A Magazine of Curiosities—A Birmingham Draughtsman—A New Number—This Week's Drug Market.

**The Operative Treatment of Inguinal Hernia in Hospital** ... ... ... ... ... ... 515

**Flies and Food** ... ... ... ... ... ... 517

Grievous Dangers and How to Prevent Them.

PAGE

**Emotion and Belief** ... ... ... ... ... ... 518

**The Editor's Letter-Box** ... — ... ... ... 519

The Criminal Law Amendment Bill.
The Royal British Nurses' Association and the College of Nursing, Limited.
Manipulative Surgery.

**The Matrons' and Sisters' Department** ... 521

The Profession of British Nurses: I. Plain Words on Registration.
Round the Hospitals.
"The Soldiers," by Archdeacon Holmes—Excellent Food in Military Hospitals—Ambitious Nurses' Memorial—"The Secret Compact" Revealed—The Sugar Ration—War Pay for Hospital Staffs.

**Some Problems of To-day** ... ... ... ... 523

I. The Future Citizen.

**Food in Wartime** ... ... ... ... ... ... 524

The Sugar Ration.

**The Patriotism of the Nurse-Training Schools** 525

XII. London Training Schools.

**Institutional Appliances** ... ... ... ... ... 526

The "Runwell" Semi-Rotary Hand Pump.

**What One Secretary is Thinking** ... ... 526

**Passing Events and Topics** ... ... ... ... iv

**Matrons' and Sisters' Appointments** ... ... iv

**The Institutional Worker** ... ... (Suppl.) 1

The Service of Patients' Dinners.

